# ^137^Cs and isotopic ratios of Pu and U in lichens and mosses from Russian Arctic areas

**DOI:** 10.1007/s11356-023-27795-4

**Published:** 2023-05-29

**Authors:** Paul Dutheil, Jussi Paatero, Ilia Rodushkin, Timo Sundström, Ari-Pekka Leppänen, Susanna Salminen-Paatero

**Affiliations:** 1 Department of Chemistry, P.O. Box 55, FI-00014 University of Helsinki, Finland; 2 Present Address: Department of Radiation Safety and Security, Paul Scherrer Institute, Forschungsstrasse 111, CH-5232 Villigen PSI, Switzerland; 3 Finnish Meteorological Institute, P.O. Box 503, Helsinki, Finland; 4 ALS Scandinavia AB, Aurorum 10, S-97775 Luleå, Sweden; 5 Radiation and Nuclear Safety Authority-STUK, Lähteentie 2, 96460 Rovaniemi, Finland

**Keywords:** Plutonium, Uranium, Isotopic ratios, Nuclear contamination, Environmental radioactivity, Arctic

## Abstract

Knowledge of past anthropogenic sources of radionuclide contamination in Russian Arctic areas is important to assess the radioecological situation of these less-studied regions. Therefore, we investigated the sources of radionuclide contamination in Russian Arctic in the 1990s. Lichen and moss samples were collected from 1993 to 1996 in Kola Peninsula, Franz Josef Land, and few other locations. The activity concentration of ^137^Cs was determined from the archived samples by gamma spectrometry in 2020. After radiochemical separation of Pu and U isotopes from the lichens and mosses, mass ratios ^240^Pu/^239^Pu, ^234^U/^238^U, ^235^U/^238^U, and ^236^U/^238^U were determined by mass spectrometry. ^137^Cs activity concentrations at the sampling date were found to vary from 3.1 ± 1.4 (Inari, Finnish-Russian border) to 303 ± 7 (Kola Peninsula) Bq/kg. The ranges of isotopic ratios were 0.0592 ± 0.0007 to 0.253 ± 0.082 for ^240^Pu/^239^Pu, (4.89 ± 3.91) × 10^−5^ to (6.86 ± 0.04) × 10^−5^ for ^234^U/^238^U, 0.0072104(21) to 0.007376(41) for ^235^U/^238^U, and from below 1 × 10^−7^ to (2.65 ± 0.19) × 10^−6^ for ^236^U/^238^U, respectively. Based on the measured isotopic ratios and characteristic isotopic ratios of known contamination sources, the main Pu and U sources in the sampled lichens and mosses are global fallout, the Chernobyl accident, and possibly local nuclear activities. These results contribute to further understanding of past nuclear events and resulting nuclear contamination in Russian Arctic terrestrial areas.

## Introduction

Radioactive contamination in terrestrial environment in Russian Arctic areas originates from various anthropogenic sources. Atmospheric nuclear weapon testing in the 1950s and 1960s has produced both global and local (from tests performed at Novaya Zemlya) fallouts to the environment. It has been estimated that about 5% of the released radioactivity from the 87 atmospheric nuclear tests performed at Novaya Zemlya in 1955–1962 was deposited as local fallout (Aarkrog 1994). Nuclear activities performed at Novaya Zemlya islands during the past decades include at least atmospheric, underground, and underwater nuclear tests, a sunken nuclear submarine K-27, and dumping of liquid and solid nuclear wastes in the sea (e.g. Matishov et al. 2021). Besides these local nuclear contamination events in Arctic Russian environment, effects from global fallout, Chernobyl accident in 1986, and Fukushima accident in 2011 have given their own contributions to nuclear contamination inventory in Russian Arctic regions. Inputs from nuclear material reprocessing and production plants in Western Europe and Siberia via sea currents have also been important nuclear contamination contributors in Russian Arctic areas (Matishov et al. 1994a). In addition, submarines that have been sunken in remote Arctic sea areas are potential sources of nuclear contamination.

Radionuclide concentrations in aquatic ecosystems in sea areas nearby Novaya Zemlya have been well investigated. For instance, Russian researchers (Matishov et al. 1994a, 2020, 2021), a joint long-term Norwegian-Russian co-operation (JRNEG 1994, 1996; Strand et al. 1994; Salbu et al. 1997; Dahle et al. 2009; JNREG 2014; Gwynn et al. 2016; Heldal et al. 2018), Polish-Russian co-operation (Matishov et al. 1994b; Szczypa and Reszka 1998), and Finnish-Russian co-operation (Matishov et al. 1995, 2011; Leppänen et al. 2013) have improved knowledge about radioecology in these aquatic ecosystems. It has been found in these studies that radionuclide concentrations in aquatic environment in Russian Arctic regions have been generally at a low level, not significantly deviating from the radioactivity levels due to global fallout. According to fore mentioned monitoring studies, no leakage from radioactive pollution sources has been detected yet. Although transfer of radionuclides via aquatic food web has been considered to be relatively low at the moment (Heldal et al. 2018), radioecological monitoring work should be continued for detecting possible forthcoming leakage promptly and for improving site-specific assessments. It has been estimated that solid radioactive waste with spent nuclear fuel, dumped by the former USSR, comprises as much as 84.4% of total long-lived anthropogenic radionuclide inventory in Barents and Kara sea ecosystems in 1961–1990 (Yablokov 2001).

Compared to aquatic environment, published and publicly available data of radionuclides in terrestrial environment and ecosystems in Russian Arctic regions is even sparser. Significant challenge in radioecological research of Russian Arctic areas is limited availability of data from the past monitoring and research work. Large part of this research data is unpublished, secreted, and restricted. For example, an original article by Matishov et al. (1994b) including activity concentrations of radionuclides in soil, lichen, and moss samples from Barents Sea islands and Novaya Zemlya is no longer available, but fortunately, Szczypa and Reszka (1998) published their own dataset on the similar topic. A more recent study explored cryoconite samples from Nalli glacier, Novaya Zemlya (Miroshnikov et al. 2021).

The aim of the current work was to shed light on the anthropogenic radionuclide concentrations and contamination sources in the Russian Arctic terrestrial regions. There are large gaps in the current knowledge of radioecological situation in these areas. Lichen and mosses were selected as sample matrices for this study due to their ability to accumulate and concentrate radionuclides from the surface air, enabling their use as biomonitors of contaminants (Tuominen and Jaakkola 1973; di Lella et al. 2003; Borylo et al. 2017; Galhardi et al. 2017). Besides global and local radionuclide depositions, other factors affecting the accumulated radionuclide concentration in lichen are, e.g., lichen species, the specific sampling site, and climatic factors (Borylo et al. 2017; Galhardi et al. 2017).

The activity concentration of ^137^Cs and mass ratios ^240^Pu/^239^Pu, ^234^U/^238^U, ^235^U/^238^U, and ^236^U/^238^U were determined from lichen and moss samples collected from different locations across the Russian Arctic areas during the 1990s. The mass and activity ratio values of ^137^Cs, ^239+240^Pu and ^234,235,236,238^U were used to identify nuclear contamination sources in investigated samples. The purpose of the study was to find out whether the extensive Soviet nuclear operations in the western part of Arctic Russia would have caused an exceptional radiological situation in the terrestrial environment of the region.

## Experimental

### Sampling

Lichen and moss samples were collected in various locations around Russian Arctic areas: Yenisey estuary, Vaygach Islands and southern part of Novaya Zemlya in 1993, Franz Josef Land in 1994, and Kola Peninsula in 1993–1996 (Table 1). M.Sc. Kristina Rissanen of Radiation and Nuclear Safety Authority (STUK) collected the samples when she had an extensive collaboration with the Russian Academy of Sciences (RAS) and especially with the Murmansk Marine Biology Institute (MMBI) of RAS (Rissanen et al. 1997; Ikäheimonen et al. 1997). Most of the samples were collected during marine expeditions of the MMBI whereas the samples from Kola Peninsula were collected during joint measurement campaigns of STUK, the Laboratory of Radiochemistry, University of Helsinki, and the Institute of Radiation Hygiene, St. Petersburg, Russia. The aim of the measurement campaigns was to study the lichen-reindeer-man food chain amongst the indigenous Sami people in Kola Peninsula (Doudarev et al. 1995). In addition to Russian samples, one moss and one lichen sampled from Finnish Lapland in 1980 and 2007 were used as reference samples representing the subarctic environment outside the former Soviet Union. The identified moss family was *Polytrichum*, whilst lichen families *Cladonia*, *Cetraria*, *Comicularia*, and *Stereocaulon* were present in the samples. Parts of the samples were also mixed lichen species. After sampling, the lichens and mosses were dried, homogenised, and analysed for various radionuclides (Rissanen et al. 1995). STUK’s Regional Laboratory in Northern Finland was closed in 2015 and its radioecological sample archive was discarded. However, a small set of lichen, moss and reindeer tissue samples were stored to the joint sample archive of University of Helsinki and the Finnish Meteorological Institute (FMI) (Paatero and Salminen-Paatero 2019). These samples were utilised in this work.

**Table 1:** Sampling sites, dates, and species of the analysed samples

Sample code	Sampling area	Sampling date	Lichen or moss species
APU24/80	Apukka, Finnish Lapland	1.7.1980	*Polytrichum* sp. (moss)
AK72/07A	Raja-Jooseppi, Finnish Lapland	8.9.2007	*Cladonia stellaris* (lichen)
AK72/07C	Raja-Jooseppi, Finnish Lapland	8.9.2007	*Cladonia stellaris* (lichen)
AK131/93A	Ostrov Nokuyav, Kola Peninsula	28.7.1993	*Cladonia mitis* (lichen)
AK126/93A	Sem' Ostrovov, Kola Peninsula	11.7.1993	*Cladonia mitis* (lichen)
AK80/93	Dalniye Zelentsy, Kola Peninsula	11.7.1993	*Cetraria nivalis* (lichen)
AK429/93A	Dalniye Zelentsy, Kola Peninsula	25.9.1993	*Cladonia mitis* (lichen)
AK433/94A	Revda, Kola Peninsula	28.8.1994	*Cetraria islandica* (lichen)
AK134/96G	Kovdor, Kola Peninsula	12.6.1996	Lichen
AK141/96	Kuzreka, Kola Peninsula	12.6.1996	Mixed lichen species
AK148/96	Lake Kolvitsa, Kola Peninsula	25.7.1996	*Cladonia stellaris* (lichen)
AK154/96	Zapodnaja Litsa river, Kola Peninsula	26.7.1996	*Cladonia stellaris* (lichen)
AK156/96	Rechka Ura river, Kola Peninsula	26.7.1996	Lichen
AK463/94A	Alger Island, Franz Josef Land	18.8.1994	Moss, top part
AK463/94B	Alger Island, Franz Josef Land	18.8.1994	Moss, bottom part
AK463/94 BW	Alger Island, Franz Josef Land	18.8.1994	Moss, bottom part
AK469/94A	Kane Island, Franz Josef Land	22.8.1994	Moss, top part
AK469/94B	Kane Island, Franz Josef Land	22.8.1994	Moss, bottom part
AK470/94A	Prince Rudolf Island, Franz Josef Land	23.8.1994	Moss
AK471/94A	Jackson Island, Franz Josef Land	23.8.1994	*Cetraria nivalis* + other species
AK127/93A	Vaygach island, Russia	20.7.1993	*Cetraria* sp. (lichen)
AK128/93A	Vaygach island, Russia	20.7.1993	*Cladonia mitis* (lichen)
AK129/93A	Novaya Zemlya, Russia	23.7.1993	*Cetraria islandica* (lichen)
AK442/93A	Yenisey estuary, Russia	18.9.1993	*Cetraria islandica* (lichen)
AK444/93	Yenisey estuary, Russia	18.9.1993	*Cetraria* sp. or *Comicularia* sp. (lichen)
AK446/93	Yenisey estuary, Russia	18.9.1993	*Stereocaulon* sp. (lichen)

### Gamma measurements

Moss and lichen samples were packed in cylindrical plastic containers with a diameter of 45 or 90 mm. They were measured with a high-purity germanium (HPGe) detector Canberra GX 8021 for 3–5 days, aimed at obtaining a peak area of at least 1000 counts for ^137^Cs. Calibration of the detector was performed in the same measurement geometry as the samples using a multigamma standard solution. The relative efficiency of the detector was 40%, and the energy resolution was 1.8 keV (FWHM) at 1.33 keV. Genie 2000 Gamma Acquisition & Analysis programme (Canberra) software was used for analysing the gamma spectra.

### Separation procedure for Pu and U

After gamma measurements, some separate top and bottom parts of the same lichen or moss having small sample masses were combined before digestion, for ensuring high signals of U and Pu isotopes in later mass spectrometric measurements. The samples were ashed in an oven at 600 °C for 12–24 h. Then 40–100 ml of reverse aqua regia (conc. HNO_3_: conc. HCl in 3:1 mixture) was added to each ashed sample. Tracers of ^242^Pu and ^232^U were added to each sample as yield determinants. Wet digestion of the samples was performed on a hot plate for 6 h or longer. Sample solutions were filtered, and the solid residue was discarded. Some samples having high sample masses were divided into two or three subsamples after wet digestion, for enabling parallel analyses. Sample solutions were evaporated to dryness.

The separation method used for Pu and U was modified from Hakanen and Jaakkola (1976) and Jaegler et al. (2019). Pu and U were separated from the sample matrix and each other by anion exchange. Before loading the sample to an anion exchange column, the evaporated sample residue was dissolved in 10–40 ml of 8 M HNO_3_, and Pu was stabilised as Pu^4+^ by adding a small amount of solid NaNO_2_ and heating. After cooling to room temperature, the sample was loaded to an anion exchange column (Dowex 1 × 4, 50–100 mesh, Sigma-Aldrich), and both the load solution and the following washing solution, 30 ml of 8 M HNO_3_, were collected with the U isotopes they contained. The column was further washed with 60 ml of 10 M HCl, and then Pu was eluted with 68 ml mixture of concentrated HCl + 1 M NH_4_I (7.5:1 mixture).

Both U and Pu fractions were further purified prior to mass spectrometric measurements. Pu fraction underwent the same anion exchange procedure again, with the half of resin and reagents volumes compared to the first anion exchange separation. U fraction was loaded as 10 ml of 4 M HNO_3_ to UTEVA (Triskem International, Bruz, France) extraction chromatography column. After washing the column with further 10 ml of 4 M HNO_3_, U was eluted with 15 ml of 0.01 M HCl. Finally, the purified U and Pu fractions were processed for ICP-MS (inductively coupled plasma-mass spectrometry) measurement by evaporation and dissolution to 5% (v/v) HNO_3_ s.p. In addition to lichen and moss samples, certified reference material samples (IAEA-384 Fangataufa sediment) and blank samples were analysed in a similar way for quality assurance.

### ICP-MS

Isotopic ratios of ^240^Pu/^239^Pu, ^234^U/^238^U, ^235^U/^238^U, and ^236^U/^238^U were determined from moss and lichen samples by multicollector inductively coupled plasma mass spectrometry (MC-ICP-MS, Neptune Plus, ThermoScientific, Bremen, Germany) and inductively coupled plasma sector field mass spectrometry (ICP-SFMS, Element XR, ThermoScientific). Aridus II desolvating nebuliser system (Cetac, Omaha, NE, USA) and MC-ICP-MS interface equipped with ‘Jet’ sampler and ‘X-type’ skimmer cone was used to increase instrumental sensitivity. Instrumental conditions and measurement parameters can be found elsewhere (Rodushkin et al. 1999; Pontér et al. 2021). In order to reduce tailings from ^238^U and ^235^U on masses 236 and 234 as well as limit ^235^UH^+^ formation affecting ^236^U, the ^236^U/^238^U ratio was measured in medium resolution mode with Retarding Potential Quadrupole (RPQ) Lenses optimised for the highest abundance sensitivity with ^236^U signal measured using ion counter. The effect from remaining spectral interferences was corrected mathematically. Internal standard (in ICP-SFMS) and external calibration with bracketing isotope standard reference materials (IRMM-184 and IRMM-187, in MC-ICP-MS) were used to correct for instrumental mass bias. Standard deviation (SD) for isotopic concentrations was calculated from two independent consecutive measurements.

## Results and discussion

### ^137^Cs activity concentration in lichens and mosses

**Fig. 1:**
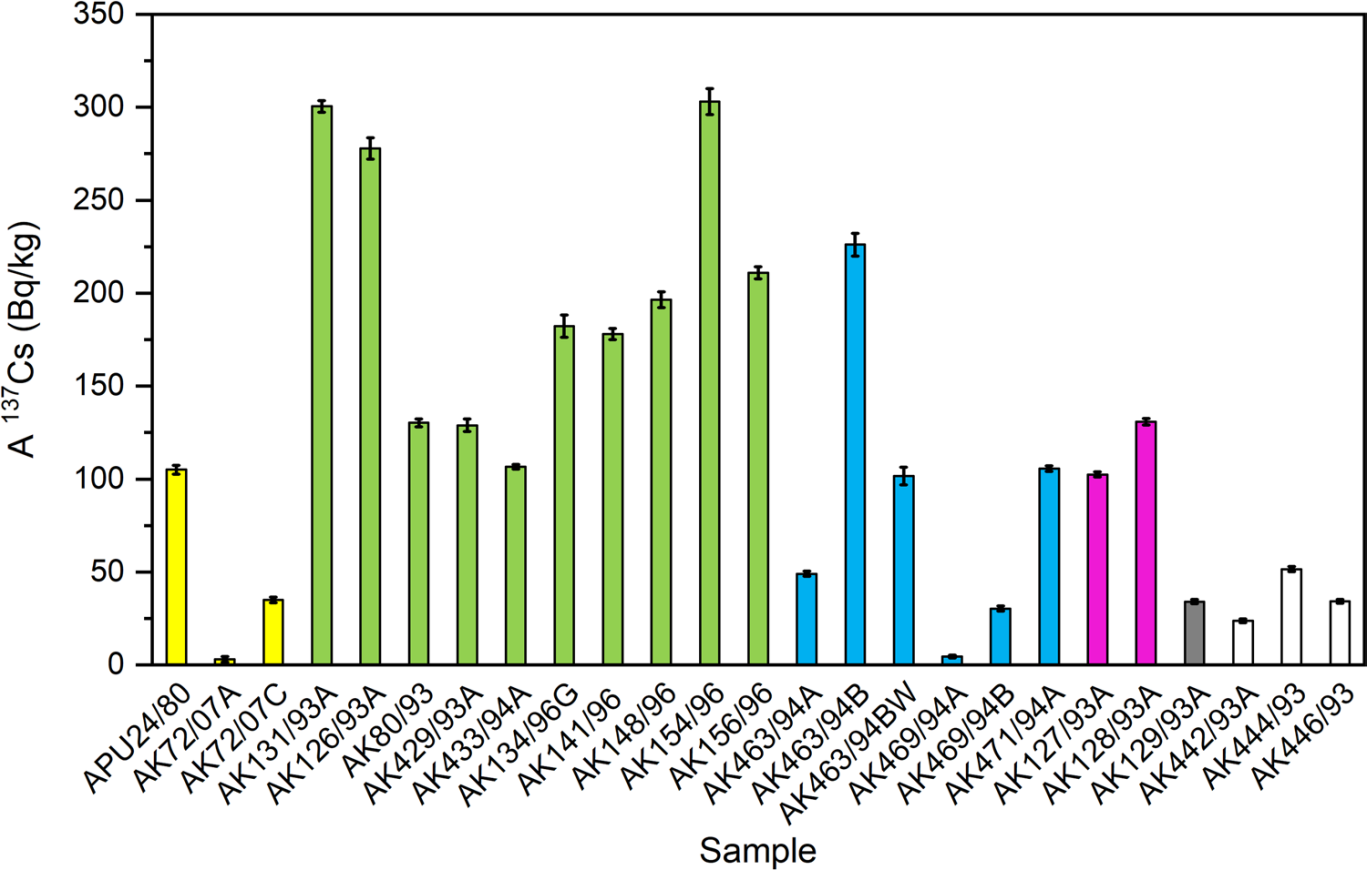
The activity concentration of ^137^Cs (Bq/kg dry weight) in lichen and moss samples from Russian and Finnish Arctic. Yellow columns: Finnish Lapland, green columns: Kola Peninsula, blue columns: Franz Josef Land, purple columns: Vaygach Island, grey column: Novaya Zemlya, and white columns: Yenisey Estuary

The activity concentration of ^137^Cs in the investigated lichen and moss samples was 107 ± 1–303 ± 7 Bq/kg in Kola Peninsula, < 3–226 ± 6 Bq/kg in Franz Josef Land, 103 ± 1 and 131 ± 2 Bq/kg in Vaygach Island, 34 ± 1 Bq/kg in Novaya Zemlya, and 24 ± 1–52 ± 1 Bq/kg in Yenisey estuary, Russia (Table 2, Fig. 1). Reference samples from Finnish Lapland had ^137^Cs activity concentrations of 3.1 ± 1.4–105 ± 2 Bq/kg. The activity concentration values (per dry weight) have been decay corrected to sampling time. Previously, the activity concentration of ^137^Cs has been determined to be 290 ± 60 Bq/kg in five lichen samples from Lovozero, Kola Peninsula, and also a low amount of ^134^Cs was then (in 1994) detected in lichens, 8.5 ± 5.6 Bq/kg (Doudarev et al. 1995). Szczypa and Reszka (1998) published very similar ^137^Cs activity concentration values, they reported 297–528 Bq/kg, 136–359 Bq/kg, 81 Bq/kg, and 5.6–395 Bq/kg for lichens and mosses of Kola Peninsula, Franz Josef Land, Vaygach Island, and Novaya Zemlya in 1991–1992, respectively. They had also determined values 359–478 Bq/kg for Dolgiy Island and 46 Bq/kg for Kolguyev Island.

**Table 2:** The activity concentration of ^137^Cs, mass ratios ^240^Pu/^239^Pu and ^235^U/^238^U, activity ratios ^239+240^Pu/^137^Cs, and ^137^Cs/ ^239+240^Pu in investigated lichens and mosses. Uncertainties for mass ratio values have been calculated from one SD (standard deviation) counting errors. 1σ error is given for the activity concentration of ^137^Cs, and the activity concentration has been decay-corrected to sampling date

Sample code	Sample mass for gamma meas. (g)	A ^137^Cs (Bq/kg)	Sample mass for digestion (g)	^240^Pu/^239^Pu	^235^U/^238^U	^234^U/^238^U	^239+240^Pu/^137^Cs	^137^Cs/^239+240^Pu
APU24/80	31	105 ± 2	10	0.192 ± 0.007	0.007376(41)	(4.9 ± 3.9) × 10^−5^	0.0052 ± 0.0006	193 ± 23
AK72/07A	12	3.1 ± 1.4	23	0.253 ± 0.031	0.0072208(7)	(5.59 ± 0.04) × 10^−5^	0.0014 ± 0.0003	720 ± 180
AK72/07C	15	35 ± 1						
AK131/93A	18	301 ± 3	18	0.211 ± 0.020	0.0072208(1)	(6.26 ± 0.01) × 10^−5^	0.0039 ± 0.0003	256 ± 20
AK126/93A	50	278 ± 6	20	0.168 ± 0.009	0.0072126(6)	(6.07 ± 0.01) × 10^−5^	0.00048 ± 0.00003	2100 ± 150
AK80/93	13	130 ± 2	20	0.204 ± 0.006	0.007212461(2)	(5.62 ± 0.01) × 10^−5^	0.00057 ± 0.00008	1760 ± 250
AK429/93A	50	129 ± 3	20	0.147 ± 0.019	Not analysed	-	0.00040 ± 0.00016	2500 ± 1000
AK433/94A	40	107 ± 1	20	0.104 ± 0.057	0.0072190(3)	(5.68 ± 0.01) × 10^−5^	0.00013 ± 0.00008	7700 ± 4600
			20	0.0592 ± 0.0007	0.0072191(1)	(5.65 ± 0.09) × 10^−5^	0.0072 ± 0.0002	138 ± 4
AK134/96G	13	182 ± 6	20	0.196 ± 0.015	0.0072133(14)	(5.58 ± 0.03) × 10^−5^	0.00068 ± 0.00009	1470 ± 190
AK141/96	36	178 ± 3	32	0.182 ± 0.009	0.0072116(24)	(5.14 ± 0.03) × 10^−5^	0.0155 ± 0.0010	62 ± 4
			20	0.180 ± 0.007	0.0072155(9)	(5.12 ± 0.03) × 10^−5^	0.0329 ± 0.0014	30 ± 1
AK148/96	40	196 ± 4	17	0.174 ± 0.005	0.0072127(5)	(5.29 ± 0.02) × 10^−5^	0.0095 ± 0.0007	106 ± 8
			20	0.176 ± 0.010	0.0072153(2)	(5.27 ± 0.02) × 10^−5^	0.0153 ± 0.0010	65 ± 4
AK154/96	32	303 ± 7	20	0.179 ± 0.014	0.0072128(2)	(5.50 ± 0.04) × 10^−5^	0.0089 ± 0.0011	112 ± 14
AK156/96	37	211 ± 3	20	0.198 ± 0.067	0.0072124(9)	(5.50 ± 0.01) × 10^−5^	0.0048 ± 0.0015	206 ± 67
AK463/94A	12	49 ± 1	20	0.178 ± 0.025	0.0072204(4)	(6.120 ± 0.003) × 10^−5^	0.0031 ± 0.0004	325 ± 41
AK463/94B	6	226 ± 6						
AK463/94BW	12	102 ± 5						
AK469/94A	15	4.5 ± 0.7	20	0.203 ± 0.024	0.0072195(6)	(6.65 ± 0.01) × 10^−5^	0.0177 ± 0.0020	56 ± 6
AK469/94B	11	30 ± 1						
AK470/94A	4	< 3	16	0.199 ± 0.026	0.0072131(14)	(6.86 ± 0.04) × 10^−5^	–	–
AK471/94A	31	106 ± 1	20	0.198 ± 0.018	0.0072113(4)	(6.22 ± 0.06) × 10^−5^	0.0087 ± 0.0015	115 ± 19
AK127/93A	15	103 ± 1	20	0.198 ± 0.003	0.0072123(10)	(5.60 ± 0.05) × 10^−5^	0.00252 ± 0.00009	398 ± 13
			20	0.208 ± 0.015	0.0072196(3)	(5.70 ± 0.03) × 10^−5^	0.0025 ± 0.0004	401 ± 65
AK128/93A	16	131 ± 2	20	< D_L_	0.0072104(21)	(5.57 ± 0.21) × 10^−5^	–	–
			19	0.183 ± 0.014	0.0072166(44)	(5.62 ± 0.16) × 10^−5^	0.00039 ± 0.00005	2600 ± 300
AK129/93A	43	34 ± 1	20	0.253 ± 0.082	0.0072104(7)	(5.86 ± 0.06) × 10^−5^	0.0015 ± 0.0004	670 ± 160
AK442/93A	18	24 ± 1	20	0.149 ± 0.026	0.0072113(2)	(5.790 ± 0.002) × 10^−5^	0.0041 ± 0.0013	245 ± 77
AK444/93	13	52 ± 1	20	< D_L_	Not analysed	–	–	–
AK446/93	15	34 ± 1	20	< D_L_	0.0072129(9)	(5.87 ± 0.02) × 10^−5^	–	–

There are no significant differences in the activity concentrations of ^137^Cs between these sampling locations, neither in the current study nor in the previous ones by Szczypa and Reszka (1998), Doudarev et al. (1995), and Rissanen et al. (1995). In the current study, only one lichen sample from Novaya Zemlya was available and its ^137^Cs activity concentration was low, 34 ± 1 Bq/kg. However, taking a closer look at Novaya Zemlya sample group of Szczypa and Reszka (1998), the difference between Chernaya Bay and other parts of Novaya Zemlya in radionuclide concentrations is clear. The activity concentration of ^137^Cs was 5.6–31 Bq/kg in lichens from Nordensheld Bay, whilst it was 312 Bq/kg in a moss and 395 Bq/kg in a lichen sample from Chernaya Bay. High concentrations of ^137^Cs and other anthropogenic radionuclides were also observed in the sediment samples collected from Chernaya Bay (Szczypa and Reszka 1998). Chernaya Bay, former Soviet military area, is the most contaminated part of the Novaya Zemlya, regarding radionuclide concentrations in environment (Matishov et al. 1994b). Based on environmental ^137^Cs activity concentrations and the absence of ^134^Cs in the environmental samples collected soon after the Chernobyl accident, it has been concluded that radioactive deposition from the Chernobyl accident to Novaya Zemlya was only minor (Szczypa and Reszka 1998; Matishov et al. 2021). According to Semenov (1993) cited by Bergman and Baklanov (1998), 30% of the ^137^Cs in Kola Peninsula soil originated from the Chernobyl accident. The activity concentration range of ^137^Cs has been summarised to be typically 100–600 Bq/kg in lichens in Kola Peninsula (Semenov 1993; Doudarev et al. 1995; Rissanen et al. 1995; Bergman and Baklanov 1998).

Although most of the anthropogenic radionuclide inventory is in the sea environment in Russian Arctic areas, here is an additional, quite recently found, radionuclide accumulator and potential source for several terrestrial environments of Arctic. Cryoconites can accumulate much higher concentrations of radionuclides and other contaminants from their environment than traditionally investigated contamination monitors, lichens, and mosses. Cryoconites are discussed here, as the existing radioactivity data for cryoconites is a welcome addition to a corresponding partly limited data for lichen and moss samples in Arctic regions. Cryoconites have been formed from fine-grained dark sediment accompanied with microbes, rocks, soot, algae, etc., which all have been carried by the wind even from far distances and deposited onto glacial surface or onto snow. Accumulation of radionuclides and other pollutants in the cryoconites have been studied, e.g., by Łokas et al. (2016, 2017, 2018, 2022), Owen et al. (2019), and Miroshnikov et al. (2021). Since the dark colour of cryoconites enhances melting of surrounding glacier or snow, release of accumulated radionuclides and other pollutants from the cryoconites into terrestrial environment can be substantial; this is a concern in the future assessments of climate change effects in the Arctic environments. Miroshnikov et al. (2021) determined the activity concentration of ^137^Cs in cryoconites from Nalli Glacier, Novaya Zemlya, in 2018, and the range was from 244 ± 4 to 8093 ± 69 Bq/kg per dry mass where the mean value was 4049 ± 33 Bq/kg. The activity concentration range varies greatly in cryoconites, depending on the exact sampling site and various other factors, as the activity concentration of ^137^Cs was observed to vary between 58 ± 2–2076 ± 20 Bq/kg (mean value 354 ± 4 Bq/kg) in the same sampling site in 2017 (Miroshnikov et al. 2021). Even higher activity concentrations of ^137^Cs have been found in cryoconites in Norway, where the range was 12.9 ± 0.8–24.5 ± 12.3 kBq/kg and average value was 17.9 ± 3.6 kBq/kg in 2018 (Łokas et al. 2022). The corresponding ranges in Svalbard cryoconites have been much lower, 89 ± 44–782 ± 99 Bq/kg, in the samples collected in 2011 and 2014 (Łokas et al. 2016). Łokas et al. (2022) concluded that most of the ^137^Cs deposition in glacier in Norway originates from the Chernobyl accident.

### ^240^Pu/^239^Pu isotope ratio in lichen and moss samples from Russian Arctic areas

**Fig. 2:**
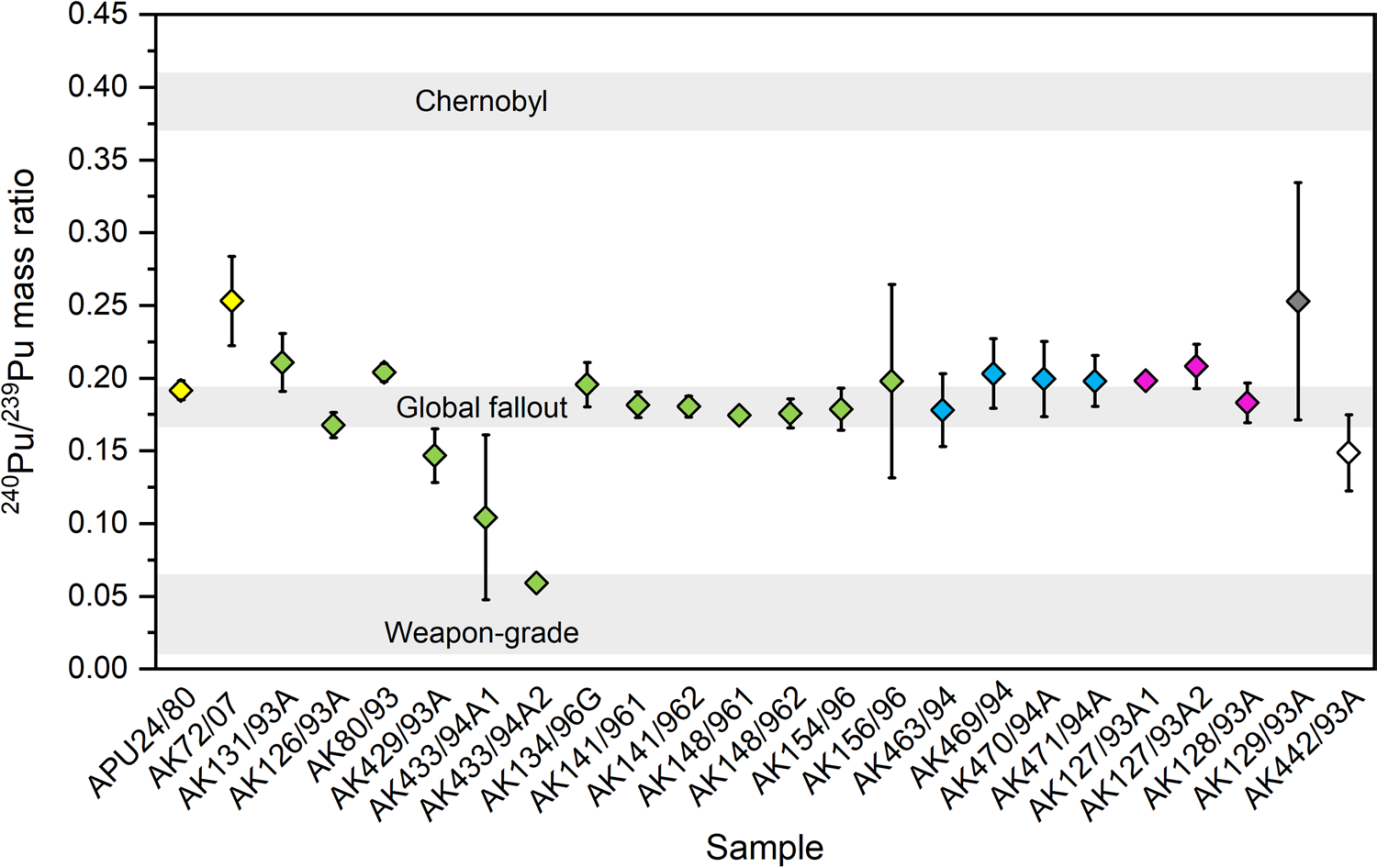
^240^Pu/^239^Pu mass ratio in lichen and moss samples from Russian Arctic areas. The error bars indicate the 1 SD counting error. Yellow dots: Finland, green dots: Kola Peninsula, blue dots: Franz Josef Land, purple dots: Vaygach Island, grey dot: Novaya Zemlya, and white dot: Yenisey estuary. The ^240^Pu/^239^Pu mass ratio value ranges for global fallout, weapons-grade deposition, and the Chernobyl-derived fallout have been published by Kelley et al. (1999), Albright et al. (1997), and Boulyga and Becker (2002), respectively

**Fig. 3:**
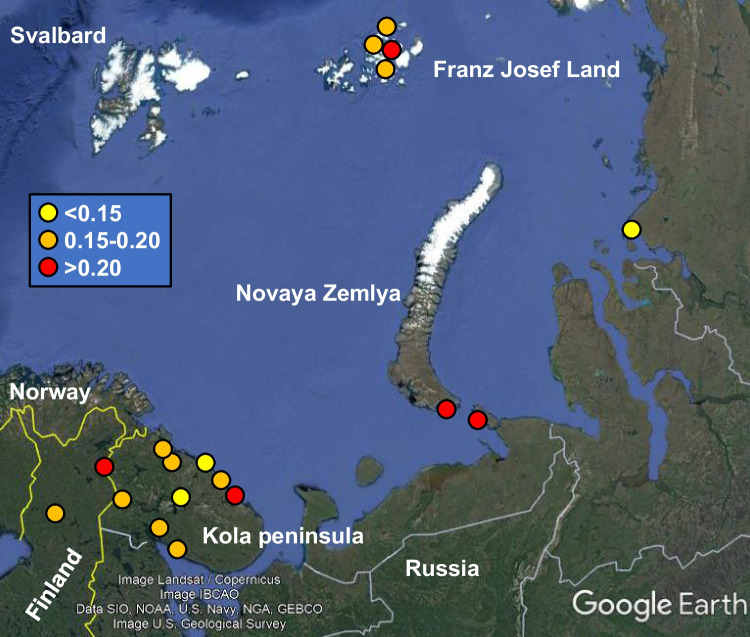
^240^Pu/^239^Pu isotope ratio in lichen and moss in the Western part of the Arctic Russia (Map: Google Earth, Google LLC)

The isotope (mass) ratio ^240^Pu/^239^Pu in all investigated lichen and moss samples varied from 0.0592 ± 0.0007 to 0.253 ± 0.082, depending on the sample and sampling site (Table 2, Figs. 2 and 3). The ^240^Pu/^239^Pu ratio varied from 0.0592 ± 0.0007 to 0.211 ± 0.020 in ten samples from Kola Peninsula, and most of the samples had the ratio close to global fallout value, which is typically assigned as ~ 0.18 (Kelley et al. 1999). Four (combined from seven samples after gamma measurements) moss and lichen samples from Franz Josef Land had the ^240^Pu/^239^Pu ratios of 0.178 ± 0.025–0.203 ± 0.024. These ratio values were close to or slightly higher than the value in global fallout. Two lichen samples from Vaygach Island were split for analysis, and the two sample halves of “AK127/93A” had the ^240^Pu/^239^Pu ratios of 0.198 ± 0.003 and 0.208 ± 0.015, whereas two sample halves of “AK128/93A” had the ratio values of below MDA and 0.183 ± 0.014. Again, the ratio values were at the same level or slightly higher than the global fallout value.

The only lichen from Novaya Zemlya had the ^240^Pu/^239^Pu ratio of 0.253 ± 0.082, which is close to the global fallout value, taking into account the uncertainty of the determined ratio value. However, in a previous study by Oughton et al. (2004), the mass ratio ^240^Pu/^239^Pu was found to vary from 0.17 to 0.28 in sediment samples from Stepovogo Fjord, Novaya Zemlya, which is close to dumped radioactive waste containers. It is therefore possible that the value obtained in this study might actually reflect the characteristic mass ratio range of the region, having an input from dumped nuclear waste. From three lichen samples collected from the Yenisey estuary, only one gave a result over the detection limit, the ^240^Pu/^239^Pu ratio being 0.149 ± 0.026 in that sample, being slightly lower than the corresponding ratio in a global fallout. In two samples (moss and lichen) from Finnish Lapland, the ^240^Pu/^239^Pu ratio was higher than in global nuclear fallout, namely 0.192 ± 0.007 and 0.253 ± 0.031.

It is extremely difficult to assess the origin of Pu in these lichen and moss samples for the cases where the mass ratio ^240^Pu/^239^Pu deviates from the global nuclear fallout value. This is due to sampling sites of this study, where multiple Pu sources may coexist and the sampling time in the 1990s was decades after the most intense atmospheric nuclear weapons testing (globally) and nuclear activities in Novaya Zemlya. By then, the originally airborne radionuclide deposition from the atmospheric nuclear emissions in the surface air has been mainly settled to ground (Hirose and Povinec 2015) and onto surfaces of lichens and mosses. The biological half-lives of radionuclides are short compared to their physical half-lives, and eventually, the lichens and mosses return the absorbed radionuclides into soil for possible remobilisation and further recirculation. In addition, the diverse and partly hidden history of nuclear operations in Novaya Zemlya complicates the estimation of Pu sources in terrestrial environment of these Arctic regions.

Lower ^240^Pu/^239^Pu values may indicate traces from weapons-grade Pu, whose mass ratio ^240^Pu/^239^Pu has been estimated to be ≤ 0.07 (Albright et al. 1997). Most clearly two subsamples of ‘AK433/94A’ and probably ‘AK429/93A’ and ‘AK442/93A’ as well have ^240^Pu/^239^Pu mass ratios representing mixture of weapons-grade Pu and e.g. Pu from global fallout, the Chernobyl accident, or other Pu source. These samples with a low ^240^Pu/^239^Pu mass ratio were all collected from Russian continent, Revda (Kola Peninsula), Dalniye Zelentsy (Kola Peninsula), and Yenisey estuary, respectively. Similarly, low ^240^Pu/^239^Pu mass ratios have been found from cryoconites sampled in Norway and Svalbard (Łokas et al. 2019, 2022). Transportation of weapons-grade Pu from Novaya Zemlya has been suggested to be Pu source in these samples (Łokas et al. 2019, 2022).

The sample “AK433/94A”from Revda had the lowest ^240^Pu/^239^Pu ratio value in this study, 0.0592 ± 0.0007, and the sampling site is located approximately 30 km from Kuel'por apatite mine near Kirovsk, Russia, where underground ‘peaceful nuclear explosions’ (PNEs) for ore fracturing were performed in 1972 and again in 1984 (Nordyke 1996; IAEA 2004). Explosion ‘Dnepr-1’ on 4^th^ September 1972 had a yield of 2.1 kilotons, detonation depth was 130 m, and the emissions from the explosion are claimed to be fully contained in the underground tunnel, only natural background radioactivity level existing in the surrounding environment (IAEA 2004). Two explosions of ‘Dnepr-2’ were executed in the same place on 27^th^ August 1984, both bombs having yields of 1.7 kilotons and detonation depth of 160 m (IAEA 2004). According to IAEA (2004), these two later explosions produced fully contained emissions and the area has only natural background radioactivity level. However, some other references (e.g. Nordyke 1996; Bergman and Baklanov 1998) report about leakage of emission products from both ‘Dnepr-1’ and ‘Dnepr-2’ explosions. Even though the radioactivity level in the surrounding environment has been evaluated as normal and activity concentration of ^137^Cs in soil, lichen and moss samples was 6–60 Bq/kg, exceptionally high ^137^Cs concentrations have been found as well, reaching 320 Bq/kg (Evseev et al. 2013). Bomb debris has been mixed with rock melt and vitrified in the underground tunnels, and 90% of it has been considered both physically and chemically stable in its repository, with low leaching (IAEA 2004). The activity level of long-lived actinides, mainly Pu, has been estimated to be from 74 to 740 Bq/g in the rock melt. In total, 396 000 tons of ore broken by these explosions were transported with conventional methods from the Kuel'por mine during 1972–1990, and the mine was shut down in 1992 (Nordyke 1996). Based on the mass ratio typical for weapons-grade Pu (and low yield explosion resulting in low neutron flux and limited production of ^240^Pu) found from Revda sample and a relatively short distance between Revda sampling site and ‘Dnepr’ explosion site, it might be possible that the origin of weapons-grade Pu would be in these ‘Dnepr’ PNEs. However, these speculations could be confirmed only by additional sampling from Revda and Kirovsk regions.

In total 3 of all 27 Pu samples had a higher mass ratio ^240^Pu/^239^Pu than the commonly agreed global fallout value of ~ 0.18, taking into account the uncertainty in the measured mass ratio values. These three samples were collected from Finnish Lapland, Kola Peninsula, and Vaygach lsland. It is not possible to identify reliably the Pu source(s) and their contributions causing the elevated mass ratio values. Plutonium in these samples may have several sources with varying and unknown mass ratios. The most probable Pu sources in addition to global fallout are the Chernobyl accident in 1986, nuclear emissions from Novaya Zemlya, and possibly even atmospheric nuclear weapons tests executed in Semipalatinsk test site in the 1960s. Łokas et al. (2022) determined several radionuclides, including Pu isotopes and ^137^Cs, from cryoconite samples taken in Norway in the 2010s. Based on the isotope ratios in cryoconites, they concluded that ^137^Cs in the samples originated mainly from the Chernobyl accident, whilst Pu is predominantly from the global fallout, although there were signatures of weapons-grade Pu from Novaya Zemlya. In another study by Łokas et al. (2019), it was found that cryoconites may contain radionuclides from a single nuclear deposition event due to non-continuous accumulation of radionuclides, resulting in different radionuclide distribution than the surrounding soil and environment, where radionuclides from a longer time period have constantly accumulated. They determined ^240^Pu/^239^Pu in soil and cryoconites from Svalbard, and as a major part of mass ratio values in soil represented the global fallout level, there were much lower mass ratio values in cryoconites, indicating possible presence of weapons-grade Pu. Wendel et al. (2013) studied air filter samples collected at sampling stations across Norway in 1957–1958 and 1961–1962. The mass ratio ^240^Pu/^239^Pu in air filters had a wide range from 0.0517 to 0.237. Based on the different mass ratios, particle analyses, and atmospheric transport modelling, the sources of Pu were concluded to be global fallout, a long-range transport from Semipalatinsk nuclear test site, and also Novaya Zemlya nuclear tests to the surface air of Norway, during the intense periods of atmospheric nuclear weapons testing.

### ^239+240^Pu/^137^Cs and ^137^Cs/^239+240^Pu activity ratios in the lichen and moss samples from the Russian Arctic

**Fig. 4:**
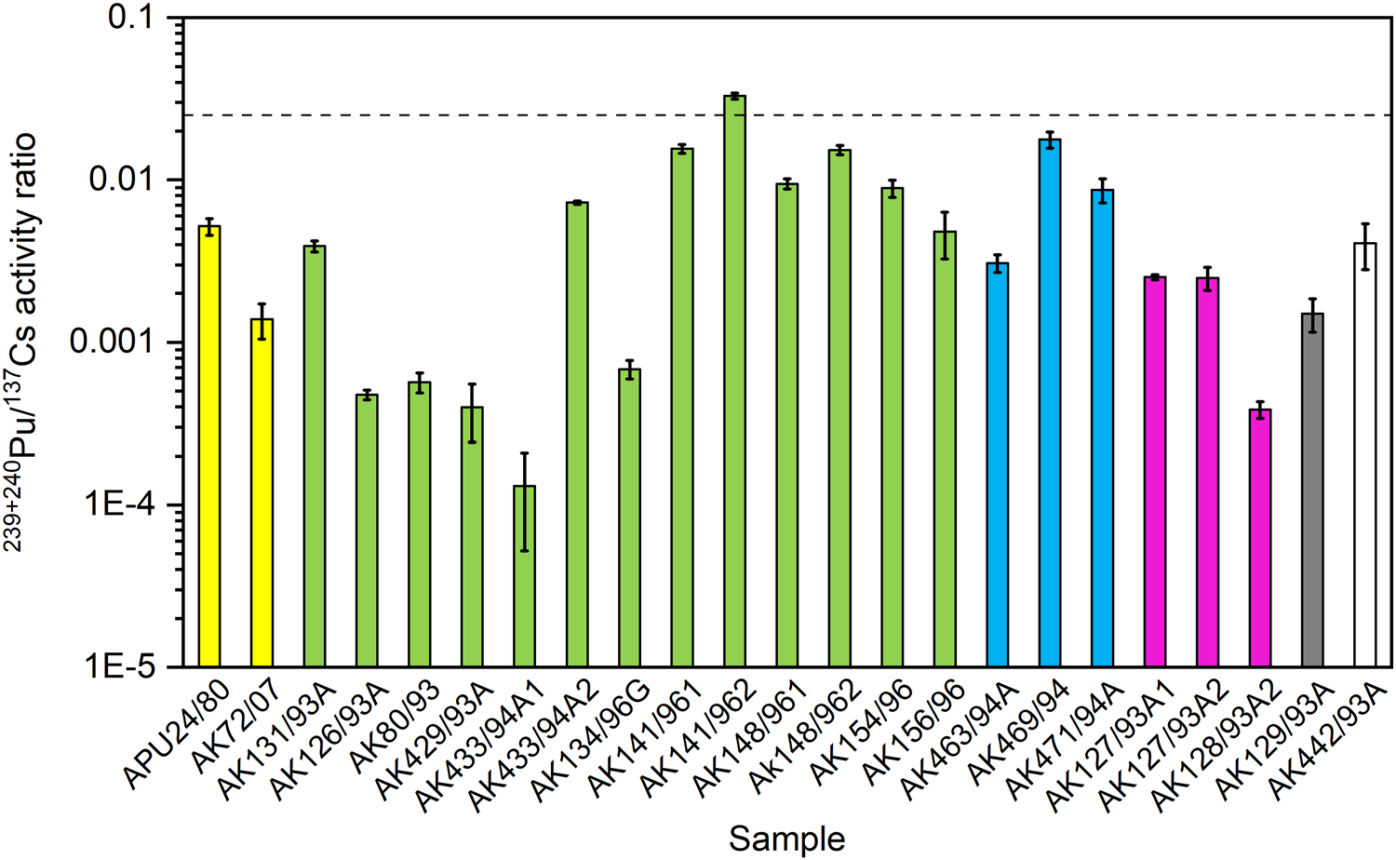
The activity ratio ^239+240^Pu/^137^Cs in lichen and moss samples. Yellow columns: Finnish Lapland, green columns: Kola Peninsula, blue columns: Franz Josef Land, purple columns: Vaygach Island, grey column: Novaya Zemlya, and white column: Yenisey Estuary. The dashed line represents the activity ratio value 0.025 in the global fallout in 1990s

The activity ratio ^239+240^Pu/^137^Cs varied from 0.00013 ± 0.00008 to 0.0329 ± 0.0014 in all investigated lichen and moss samples from Russian Arctic areas (Table 2, Fig. 4). The reverse activity ratio ^137^Cs/^239+240^Pu, which is used equally often in the literature, ranged from 30 ± 1 to 7700 ± 4600. The obtained activity ratio values have again a wide range, representing several nuclear contamination sources present in these samples.

According to de Cesare et al. (2015) and Kelley et al. (1999), the ^137^Cs/^239+240^Pu activity ratio was about 28 (i.e. ^239+240^Pu/^137^Cs would be about 0.0357) in global fallout in 2008. Another approach for calculating the ^239+240^Pu/^137^Cs activity ratio in global fallout gave the value of 0.028 (^137^Cs/^239+240^Pu being ~ 36), in Northern Hemisphere in 1999 (Łokas et al. 2022). These calculations were based on the UNSCEAR data (1982, 2000). Bossew et al. (2007) provided the third example of the ^239+240^Pu/^137^Cs activity ratio in a global fallout. They ended up to a value of 0.0180 ± 0.0024, for the 1^st^ of May, 1986, calculated further from the original data of Bunzl and Kracke (1988). The aforementioned three independently determined values for the ^239+240^Pu/^137^Cs activity ratio in a global fallout are in a good agreement. The slight variation in the ^137^Cs/^239+240^Pu ratio values amongst literature is inevitable. This is due to the range of activity concentrations in published references and constantly decreasing ratio due to the decay of ^137^Cs. Furthermore, due to their chemical differences, the respective mobility of ^239+240^Pu and ^137^Cs can be different depending on the environment. This fractionation might have affected to the activity concentrations of ^239+240^Pu and ^137^Cs in lichens and mosses of our study as well. However, quantitative effects from the possible fractionation are not known, and this possibility of occurred fractionation should be kept in mind, whilst assessing this ^137^Cs and ^239+240^Pu data. The time interval between nuclear contamination deposition and sampling time also affects the activity ratio of ^137^Cs/^239+240^Pu determined from an environmental sample. Considering all this information, we concluded that the ^239+240^Pu/^137^Cs activity ratio in global fallout was about 0.025 (and ^137^Cs/^239+240^Pu about 40) during sampling time of this study; only one of our samples had resembling activity ratio values, ‘AK141/96’ (Kuzreka, Kola Peninsula).

Compared to corresponding values in global fallout, higher ^137^Cs/^239+240^Pu (> 40 during sampling time) and lower ^239+240^Pu/ ^137^Cs (< 0.025) activity ratios were found in all samples, except the fore mentioned ‘AK141/96’ (Table 2). De Cesare et al. (2015) determined the activity ratio ^137^Cs/^239+240^Pu from the soil samples collected nearby a nuclear power plant in Italy, and they got a ratio value range of 37–58. It was concluded in their study that the ^137^Cs/^239+240^Pu ratio values in Italy were higher than the global fallout value (~ 28 in 2008) due to received contamination from the Chernobyl accident. The reverse activity ratio ^239+240^Pu/^137^Cs was 0.017–0.027, which is a bit lower than the global fallout ratio value in 2008, which was 0.0357. Even higher values for ^137^Cs/^239+240^Pu activity ratio has been determined for surface soil samples in Piora Valley, Swiss Alps, the range being 90–898 and ^239+240^Pu/^137^Cs ratio being 0.0011–0.0111 (Alewell et al. 2014). Cryoconites in Norwegian glacier had ^239+240^Pu/^137^Cs activity ratio values about 0.005 ± 0.001, ^137^Cs/^239+240^Pu ratio being about 200 (Łokas et al. 2022). These two studies named the Chernobyl accident as the main source for ^137^Cs deposition in their samples. Bossew et al. (2007) also determined the activity ratio ^239+240^Pu/^137^Cs in Chernobyl fallout, the value being 6.6 × 10^−6^, from aerosol measurements performed in Salzburg and reported originally by Irlweck et al. (1993).

The phenomenon of ^137^Cs mainly originating from Chernobyl whilst ^239+240^Pu mainly originating from global fallout in environmental samples has been observed in several studies, e.g., in Swiss Alps (Alewell et al. 2014), Norwegian glaciers (Łokas et al. 2022), and also in Finnish Lapland (Salminen-Paatero et al. 2020). The radioactive deposition from global fallout was more uniform than from the Chernobyl accident. Furthermore, the deposition area of ^137^Cs from the Chernobyl accident was much wider than that of ^239+240^Pu and other transuranium radionuclides. For example, the activity concentration of ^137^Cs in surface air of Rovaniemi, Finnish Lapland, peaked in April–June 1986, reaching a value of 1294 ± 7 μBq/m^3^, but activity concentrations of Pu isotopes ^238^Pu, ^239+240^Pu, and ^241^Pu were below detection limits in the same sample (Salminen-Paatero et al. 2019, 2020). However, it was possible to determine ^240^Pu/^239^Pu mass ratio from this sample, and the value was 0.278 ± 0.093, indicating the presence of Chernobyl-derived Pu in this pooled air filter sample, which was collected during three months after the accident happened (Salminen-Paatero et al. 2020). In the same study, ^239+240^Pu/^137^Cs ratio in the surface air could not be determined for April–June 1986 due to ^239+240^Pu having activity concentration below detection limit. During the sampling time of this study, i.e. mid-1990s, the ^239+240^Pu/^137^Cs activity ratio was 0.0393 ± 0.0038 in surface air in Rovaniemi in 1991–1995 (Salminen-Paatero et al. 2020). This value was slightly higher than in the global fallout at the same time. Before and after this sampling period, the corresponding ratio values were lower, 0.0014 ± 0.0001 in 1987–1990 and 0.0106 ± 0.0010 in 1996–2000.

Most likely higher ^137^Cs/^239+240^Pu and lower ^239+240^Pu/ ^137^Cs activity ratios, compared to global fallout values, in the studied lichen and moss samples originate from Chernobyl-derived deposition in Russian and Finnish Arctic areas. However, possibility of influences from past nuclear activities in Novaya Zemlya cannot be excluded.

It should be noted that two subsamples of sample ‘433/94A’ (Revda, Kola Peninsula) have different values for ^240^Pu/^239^Pu mass ratio and ^239+240^Pu/^137^Cs activity ratio. Whereas the mass ratio value ^240^Pu/^239^Pu is typical for weapons-grade Pu in one subsample and for a mixture of global fallout and weapons-grade unexploded material in another subsample, the difference between two subsamples is more obvious in ^239+240^Pu/^137^Cs activity ratio. The first subsample (having ^240^Pu/^239^Pu mass ratio 0.104 ± 0.057 and ^239+240^Pu/^137^Cs activity ratio 0.00013 ± 0.00008) has high uncertainties in the isotope ratio values. Due to unknown radioanalytical or mass spectrometry-related reasons, the signals of Pu isotopes in this sample were much weaker in ICP-MS measurements compared to another subsample, resulting in high standard deviations in the determined Pu isotope concentrations. The second subsample (having ^240^Pu/^239^Pu mass ratio 0.0592 ± 0.0007 and ^239+240^Pu/^137^Cs activity ratio 0.0072 ± 0.0002) produced better counting statistics in ICP-MS determination of Pu isotopes and the uncertainties of isotope ratios are therefore lower. Thus, the main reason for different isotopic and activity ratios between two subsamples of ‘433/94A’ is higher uncertainty in the values of the first subsample, and the values of the second subsamples are then more reliable and informative.

### ^234^U/^238^U, ^235^U/^238^U, and ^236^U/^238^U isotope ratios in lichen and moss samples

**Fig. 5:**
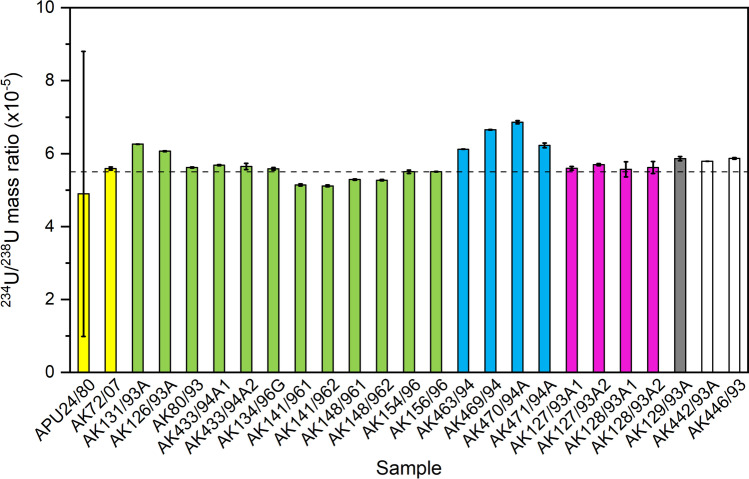
The mass ratio ^234^U/^238^U in lichen and moss samples. Yellow columns: Finnish Lapland, green columns: Kola Peninsula, blue columns: Franz Josef Land, purple columns: Vaygach Island, grey column: Novaya Zemlya, and white columns: Yenisey Estuary. The dashed line represents the mass ratio value 0.000055 in natural uranium

The mass ratio ^234^U/^238^U varied from (4.89 ± 3.91) × 10^−5^ to (6.86 ± 0.04) × 10^−5^ in lichen and moss samples from Russian and Finnish Arctic (Table 2, Fig. 5). It is known that the mass ratio ^234^U/^238^U has a wide range of values both in terrestrial and aquatic environments, due to isotopic fractionation of U, and higher mobility of ^234^U compared to ^238^U due to alpha recoil effects and nuclear decay induced oxidation of ^234^U (Suksi et al. 2006). In natural U, the mass ratio ^234^U/^238^U is 0.000055. The mass ratio values of lichen and moss samples of this study are so close to the natural U level that no traces from isotopic fractionation are visible, and the isotopes ^234^U and ^238^U are in a secular equilibrium in the samples.

**Fig. 6:**
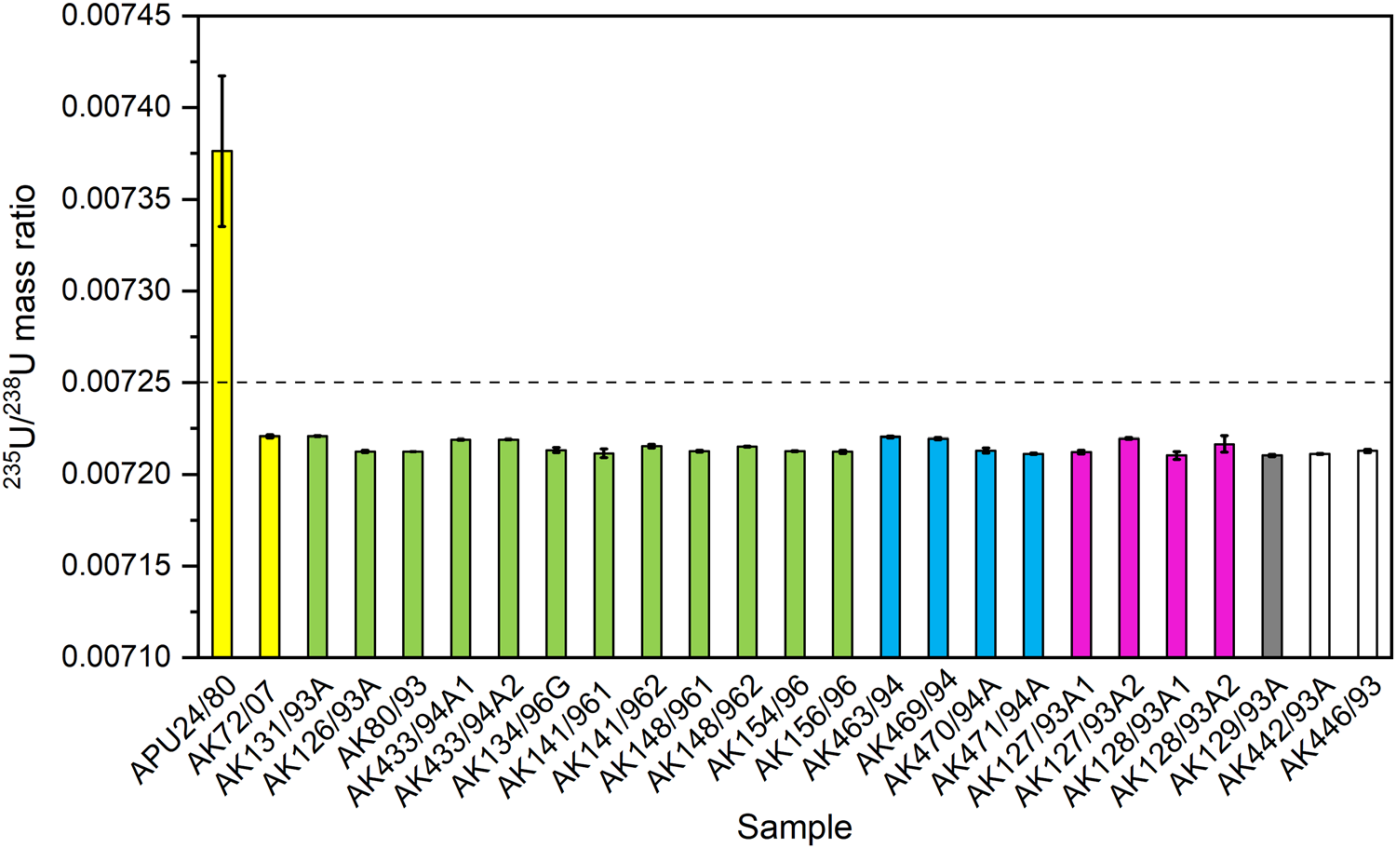
The mass ratio ^235^U/^238^U in lichen and moss samples. Yellow columns: Finnish Lapland, green columns: Kola Peninsula, blue columns: Franz Josef Land, purple columns: Vaygach Island, grey column: Novaya Zemlya, and white columns: Yenisey Estuary. The dashed line represents the mass ratio value 0.00725 in natural uranium

The mass ratio ^235^U/^238^U varied from 0.0072104(21) to 0.00738(4) in investigated samples from Russian and Finnish Arctic (Table 2, Fig. 6). The corresponding ratio in natural uranium is 0.00725*,* in emissions from the Chernobyl accident 0.00745–0.01006 (Boulyga et al. 2000) and in global fallout 0.0055–0.0097 (Bellis et al. 2001). Except the sample ‘APU24/80’ (Rovaniemi, Finland), all our samples had a ^235^U/^238^U ratio value lower than natural value, which indicates that there are no signs of enriched U in these samples. It can’t be excluded that isotopic fractionation processes have affected on the amount of ^235^U during sample preparation and determination by ICP-MS, resulting in these mass ratio values slightly lower than of natural U value. Similar phenomenon was observed by Golubev et al. (2005), when they determined ^235^U/^238^U mass ratio from digested lichen and insoluble residue after digestion. The digested part of the lichen had a ratio value 0.0050, whereas the insoluble residue had the ratio value corresponding to natural U, 0.0072. The lichen sample ‘APU24/80’ of this study had the highest ^235^U/^238^U value, 0.00738(4), and it dates back to 1980. The sample may contain traces of highly enriched uranium from the atmospheric nuclear tests. The last atmospheric nuclear test was conducted in 1980. If the biological half-life of uranium in lichen is similar to that of plutonium, two years (Paatero et al. 1998), then in the 1990s, after 5–6 biological half-lives, it would be quite impossible to detect these traces. However, ^235^U/^238^U is not a very good indicator for observing nuclear events in the environment, compared to e.g. ^236^U/^238^U mass ratio.

The ^236^U/^238^U mass ratio was measured from the samples as well. However, due to very low concentration of ^236^U in most of the samples and interferences from ^235^U, ^238^U, and polyatomic ^235^UH^+^ species, which cannot be prevented by chemical separations (Bu et al. 2017), the ratio could be reliably determined only from seven samples. The samples were all collected from Kola Peninsula and Franz Josef Land (Table 3). The ^236^U/^238^U mass ratio varied from (1.35 ± 0.13) × 10^−6^ to (1.93 ± 0.07) × 10^−6^ in Kola Peninsula samples and from (4.98 ± 0.80) × 10^−7^ to (2.65 ± 0.19) × 10^−6^ in Franz Josef Land samples. As the isotope ^236^U is mainly of anthropogenic origin, the natural ^236^U/^238^U ratio in environment was extremely low before any atmospheric nuclear weapons tests and other nuclear material emissions occurred, having values from 10^−14^ to 10^−11^ (Steier et al. 2008). Sakaguchi et al. (2009) determined ^236^U/^238^U from soil samples having only global fallout as a contamination source, in Ishikawa prefecture, Japan, and the ratio ranged from 1.85 × 10^−8^ to 1.09 × 10^−7^. Even higher values, on 10^−6^ level, have been determined from environmental samples containing global fallout as the only nuclear contamination source (Ketterer et al. 2013; Quinto et al. 2013). At this ratio level, the nuclear contamination source might be either pure global fallout, or a mixture of global fallout and local contamination, and it is helpful to have other isotopic ratios available in identification of contamination origin. ^236^U/^238^U was (8.0 ± 0.4) × 10^−7^ and (1.48 ± 0.01) × 10^−6^ in soil samples contaminated by both global fallout and the Chernobyl accident (Steier et al. 2008). They were collected from 0.5 mm and 15 mm depths, respectively, in Salzburg, Austria. ^236^U/^238^U ratio ranged from 10^–5^ to 10^–3^, in soil samples taken from relocation zone in Belarus, 7–20 km from Chernobyl NPP, and which were contaminated by the Chernobyl accident (Boulyga and Becker 2001). Based on these determined and estimated ^236^U/^238^U mass ratios for different contamination sources, the values obtained for our samples from Kola Peninsula and Franz Josef Land represent most likely contamination from global fallout. This conclusion is supported by the corresponding ^240^Pu/^239^Pu mass ratios of the same samples.

**Table 3:** ^236^U/^238^U mass ratio in lichen and moss samples from Russian Arctic

Sample code	Location	^236^U/^238^U
AK148/96	Lake Kolvitsa, Kola Peninsula	(1.81 ± 0.34) × 10^−6^
		(1.86 ± 0.16) × 10^−6^
AK154/96	Zapodnaja Litsa river, Kola Peninsula	(1.93 ± 0.07) × 10^−6^
AK156/96	Rechka Ura river, Kola Peninsula	(1.35 ± 0.13) × 10^−6^
AK463/94 A	Alger Island, Franz Josef Land	(4.98 ± 0.80) × 10^−7^
AK470/94 A	Prince Rudolf Island, Franz Josef Land	(2.65 ± 0.19) × 10^−6^
AK471/94 A	Jackson Island, Franz Josef Land	(2.28 ± 0.53) × 10^−6^

Previously discussed ^236^U/^238^U ratios for different contamination sources were determined from soil, lichen, and moss samples. However, it has to be emphasised that ^236^U/^238^U signal may be diluted in these sample types during time between deposition and sampling, due to circulation of natural U in terrestrial environment. As a consequence, the determined ^236^U/^238^U mass ratio values from soil and vegetation samples may be lower than they originally were during the time of deposition. In their recent study, Wallner et al. (2022) analysed archived air filter samples collected in Vienna and Salzburg, Austria, in 1962–1966. During that time period, only global fallout was considered as nuclear contamination source for these samples. They found unusually high ^236^U/^238^U ratio values for the air filters, compared to previously determined values for soil samples, the highest ^236^U/^238^U ratio value being (1.19 ± 0.31) × 10^−5^ in a pooled air filter sample collected in spring 1964. The authors consider that the ^236^U/^238^U ratio would have been even higher in a similar air filter sample in spring 1963, time of maximum deposition from atmospheric nuclear weapon testing, but unfortunately they did not have an air filter sample from that period. The observations by Wallner et al. (2022) show that when comparing the ^236^U/^238^U mass ratio values in environmental samples, the sample type, and possibilities for the alteration of the isotopic composition in the sample between the time of deposition and sampling time (as is the case with soil, peat, lichen, etc.) should be carefully considered.

## Conclusions

The activity concentration of ^137^Cs, the activity ratio ^137^Cs/^239+240^Pu (and ^239+240^Pu/^137^Cs), the mass ratios ^240^Pu/^239^Pu, ^234^U/^238^U, ^235^U/^238^U, and ^236^U/^238^U were determined from lichen and moss samples collected from different locations in the Russian Arctic areas. The aim was to improve the awareness of the radioecological situation of these areas, because part of the older environmental radioactivity data is no longer publicly available, and some areas investigated in our study have not been previously surveyed. As a result, the sources for ^137^Cs, Pu, and U were found to be predominantly global fallout from atmospheric nuclear weapon testing, but also the Chernobyl accident and possible local nuclear sources have affected to anthropogenic radionuclide deposition in the Russian Arctic regions. No signs of serious nuclear contamination can be seen in the radionuclide concentrations and isotopic ratios, despite the huge volume of nuclear activities that have occurred in the investigated area. However, as an interesting detail, a weapons-grade ^240^Pu/^239^Pu mass ratio was detected in a lichen sample from Revda, Kola Peninsula, close to the location where peaceful nuclear detonations were conducted. This indicates the need for a radioecological mapping of Kola Peninsula with additional samples and radionuclide analysis with nuclear particle characterisation, to identify the nuclear contamination source behind the exceptional mass ratio value.

## Data Availability

All data produced in this study are incorporated into the article.

## References

[CR1] Aarkrog A (1994). Radioactivity in polar regions - main sources. J Environ Radioact.

[CR2] Albright D, Berkhout F, Walker W (1997). Plutonium and highly enriched uranium 1996 - world inventories, capabilities and policies. Stockholm International Peace Research Institute SIPRI.

[CR3] Alewell C, Meusburger K, Juretzko G, Mabit L, Ketterer ME (2014). Suitability of 239+240Pu and 137Cs as tracers for soil erosion assessment in mountain grasslands. Chemosphere.

[CR4] Bellis D, Ma R, Bramall N, McLeod CW, Chapman N, Satake K (2001). Airborne uranium contamination as revealed through elemental and isotopic analysis of tree bark. Environ Pollut.

[CR5] Bergman R, Baklanov A (1998) Radioactive sources of main radiological concern in the Kola-Barents region. FOA-B—98–00343–861—SE, July 1998, Defence Research Establishment, Division of NBC Defence, Umeå, Sweden

[CR6] Borylo A, Romanczyk G, Skwarzec B (2017). Lichens and mosses as polonium and uranium biomonitors on Sobieszewo Island. J Radioanal Nucl Chem.

[CR7] Bossew P, Lettner H, Hubmer A, Erlinger C, Gastberger M (2007). Activity ratios of 137Cs, 90Sr and 239+240Pu in environmental samples. J Environ Radioact.

[CR8] Boulyga S, Becker J (2001). Determination of uranium isotopic composition and 236U content of soil samples and hot particles using inductively coupled plasma mass spectrometry. Fresenius J Anal Chem.

[CR9] Boulyga S, Becker J (2002). Isotopic analysis of uranium and plutonium using ICP-MS and estimation of burn-up of spent uranium in contaminated environmental samples. J Anal at Spectrom.

[CR10] Boulyga SF, Becker JS, Matusevitch JL, Dietze H-J (2000). Isotope ratio measurements of spent reactor uranium in environmental samples by using inductively coupled plasma mass spectrometry. Int J Mass Spectrom.

[CR11] Bu W, Zheng J, Ketterer ME, Hu S, Uchida S, Wang X (2017). Development and application of mass spectrometric techniques for ultra-trace determination of ^236^U in environmental samples - a review. Anal Chim Acta.

[CR12] Bunzl K, Kracke W (1988). Cumulative deposition of 137Cs, 238Pu, 239,240Pu and 241Am from global fallout in soils from forest, grassland and arable land in Bavaria (FRG). J Environ Radioact.

[CR13] Dahle S, Savinov V, Carroll JL, Vladimirov M, Ivanov G, Valetova NK, Gaziev YI, Dunaev GE, Kirichenko ZA, Nikitin AI, Petrenko GI, Polukhina AM, Kalmykov S, Aliev R, Sabodina M (2009). A return to the nuclear waste dumping sites in the Bays of Novaya Zemlya. Radioprotection.

[CR14] de Cesare M, Tims SG, Fifield LK (2015). Uranium comparison by means of AMS and ICP-MS and Pu and ^137^Cs results around an Italian Nuclear Power Plant. EPJ Web Conf.

[CR15] Di Lella LA, Frati L, Loppi S, Protano G, Riccobono F (2003). Lichens as biomonitors of uranium and other trace elements in an area of Kosovo heavily shelled with depleted uranium rounds. Atmos Environ.

[CR16] Doudarev AA, Bylinkin SV, Chugunov VV, Miretsky GI, Popov AO, Rahola T, Jaakkola T, Rissanen K, Illukka E, Suomela M, Tillander M (1995) The radioecological situation in the reindeer herding area of the Kola Peninsula. In: Strand P, Cooke A (eds.). Environmental Radioactivity in the Arctic. Proceedings of the Second International Conference on Environmental Radioactivity in the Arctic, Oslo, Norway, August 1995. Statens Strålevern, Østerås, Norway, 282–286

[CR17] Evseev AV, Telelekova AD, Sedova NB (2013) Radionuclides in the Kola Peninsulas’ geosystems. In: Kalmykov SN, Petrov VG, Romanchuk AY (Eds.), Russian-Nordic Symposium on Radiochemistry, Moscow Russia, 21–24 October 2013, Abstracts, p 115

[CR18] Galhardi JA, García-Tenorio R, Díaz Francés I, Bonotto DM, Marcelli MP (2017). Natural radionuclides in lichens, mosses and ferns in a thermal power plant and in an adjacent coal mine area in southern Brazil. J Environ Radioact.

[CR19] Golubev AV, Golubeva VN, Krylov NG, Kuznetsova VF, Mavrin SV, Aleinikov AYu, Hoppes WG, Surano KA (2005). On monitoring anthropogenic airborne uranium concentrations and ^235^U/^238^U isotopic ratio by Lichen - bio-indicator technique. J Environ Radioact.

[CR20] Gwynn JP, Nikitin A, Shershakov V, Heldal HE, Lind B, Teien H-C, Lind OC, Sidhu RS, Bakke G, Kazennov A, Grishin D, Fedorova A, Blinova O, Sværen I, Liebig PL, Salbu B, Wendel CC, Strålberg E, Valetova N, Petrenko G, Katrich I, Logoyda I, Osvath I, Levy I, Bartocci J, Pham MK, Sam A, Nies H, Rudjord AL (2016). Main results of the 2012 Joint Norwegian-Russian expedition to the dumping sites of the nuclear submarine K-27 and solid radioactive waste in Stepovogo Fjord, Novaya Zemlya. J Environ Radioact.

[CR21] Hakanen M, Jaakkola T (1976) Distribution of fallout plutonium in reindeer. In: Miettinen JK. (Ed.). Radioactive foodchains in the subartic environment (7th ed.), Contract No CH E (11–1)-3011 from US ERDA, Department of Radiochemistry, University of Helsinki. Paper No. 77

[CR22] Heldal HE, Bogstad B, Dolgov AV, Gwynn JP, Teien H-C (2018). Observations of biota in Stepovogo Fjord, Novaya Zemlya, a former dumping site for radioactive waste. Polar Biol.

[CR23] Hirose K, Povinec P (2015). Sources of plutonium in the atmosphere and stratosphere-troposphere mixing. Sci Rep.

[CR24] IAEA (2004). Nuclear explosions in the USSR: The North Test Site, Reference material. Version 4, December 2004. The Division of Nuclear Safety and Security.

[CR25] Ikäheimonen TK, Rissanen K, Matishov DG, Matishov GG (1997). Plutonium in fish, algae, and sediments in the Barents, Petshora and Kara Seas. Sci Tot Env.

[CR26] Irlweck K, Khademi B, Henrich E, Kronraff R (1993). 239(240),238Pu, 90Sr, 103Ru and 137Cs concentrations in surface air in Austria due to dispersion of Chernobyl releases over Europe. J Environ Radioact.

[CR27] Jaegler H, Pointurier F, Diez-Fernandez S, Gourgiotis A, Isnard H, Hayashi S, Tsuji H, Onda Y, Hubert A, Laceby JP, Evrard O (2019). Reconstruction of uranium and plutonium isotopic signatures in sediment accumulated in the Mano Dam reservoir, Japan, before and after the Fukushima nuclear accident. Chemosphere.

[CR28] Gwynn JP, Nikitin AI, JNREG (2014). Investigation into the radioecological status of the Stepovogo Fjord. The dumping site of the nuclear submarine K-27 and solid radioactive waste. Results from the 2012 research cruise. Joint Norwegian-Russian Expert Group.

[CR29] JRNEG (1994). Radioactive contamination at dumping sites for nuclear waste in the Kara Sea. Results from the Russian-Norwegian 1993 Expedition to the Kara Sea.

[CR30] JRNEG (1996). Dumping of radioactive waste and investigation of radioactive contamination in the Kara Sea. Results from 3 years of investigations (1992–1994) in the Kara Sea.

[CR31] Kelley JM, Bond LA, Beasley TM (1999). Global distribution of Pu isotopes and Np-237. Sci Total Environ.

[CR32] Ketterer ME, Groves AD, Strick BJ, Asplund CS, Jones VJ (2013). Deposition of ^236^U from atmospheric nuclear testing in Washington state (USA) and the Pechora region (Russian Arctic). J Environ Radioact.

[CR33] Leppänen AP, Kasatkina N, Vaaramaa K, Matishov GG, Solatie D (2013). Selected anthropogenic and natural radioisotopes in the Barents Sea and off the western coast of Svalbard. J Environ Radioact.

[CR34] Łokas E, Zaborska A, Kolicka M, Różycki M, Zawierucha K (2016). Accumulation of atmospheric radionuclides and heavy metals in cryoconite holes on an Arctic glacier. Chemosphere.

[CR35] Łokas E, Wachniew P, Jodłowski P, Gasiorek M (2017). Airborne radionuclides in the proglacial environment as indicators of sources and transfers of soil material. J Environ Radioact.

[CR36] Łokas E, Zawierucha K, Cwanek A, Szufa K, Gaca P, Mietelski JW, Tomankiewicz E (2018). The sources of high airborne radioactivity in cryoconite holes from the Caucasus (Georgia). Sci Rep.

[CR37] Łokas E, Zaborska A, Sobota I, Gaca P, Milton JA, Kocurek P, Cwanek A (2019). Airborne radionuclides and heavy metals in high Arctic terrestrial environment as the indicators of sources and transfers of contamination. Cryosphere.

[CR38] Łokas E, Wachniew P, Baccolo G, Gaca P, Janko K, Milton A, Buda J, Komędera K, Zawierucha K (2022). Unveiling the extreme environmental radioactivity of cryoconite from a Norwegian glacier. Sci Tot Env.

[CR39] Matishov GG, Matishov DG, Nazimov V (1994a) Levels and main pathways of radionuclides in the Barents and Kara Seas. Academy of Sciences of Russia, Murmansk Marine Biological Institute

[CR40] Matishov DG, Matishov GG, Szczypa E, Pavlova LG (1994). New data on radionuclide content in the Barents Sea and on the shore. Dokl Biol Sci.

[CR41] Matishov GG, Kasatkina NE, Leppänen A-P, Matishov DG, Solatie D (2011). New data on the concentration of plutonium isotopes in the sediments of the Barents Sea. Dokl Earth Sci.

[CR42] Matishov GG, Ilyin VG, Usyagina IS, Valuyskaya DA, Kirillova EE (2020). Current levels of artificial radioisotopes in biota from the Arctic continental shelf (2013–2018). Dokl Earth Sc.

[CR43] Matishov GG, Ilyin GV, Usyagina IS (2021). Main sources of ^134^Cs in the Barents and Kara Seas (1960–2020). Dokl Earth Sc.

[CR44] Matishov DG, Matishov GG, Rissanen K (1995) Peculiarities of radionuclides’ accumulation in benthic organisms and fish in the Barents and Kara Seas. In: Cooke A. (Ed.). Environmental radioactivity in the Arctic, 2nd international conference on environmental radioactivity in the Arctic, Oslo, Norway, 21–25 Aug 1995, 233–237

[CR45] Miroshnikov A, Flint M, Asadulin E, Aliev R, Shiryaev A, Kudikov A, Khvostikov V (2021). Radioecological and geochemical peculiarities of cryoconite on Novaya Zemlya glaciers. Sci Rep.

[CR46] Nordyke MD (1996) The Soviet Program for Peaceful Uses of Nuclear Explosions. UCRL-ID-124410, Contract W-7405-ENG-48, for US Department of Energy, Lawrence Livermoore National Laboratory, USA

[CR47] Oughton DH, Skipperud L, Fifield LK, Cresswell RG, Salbu B, Day P (2004). Accelerator mass spectrometry measurement of ^240^Pu/^239^Pu isotope ratios in Novaya Zemlya and Kara Sea sediments. Appl Radiat Isotopes.

[CR48] Owen PN, Blake WH, Millward GE (2019). Extreme levels of fallout radionuclides and other contaminants in glacial sediment (cryoconite) and implications for downstream aquatic ecosystems. Sci Rep.

[CR49] Paatero J, Jaakkola T, Kulmala S (1998). Lichen (Sp. Cladonia) as deposition indicator for transuranium elements investigated with the Chernobyl fallout. J Environ Radioact.

[CR50] Paatero J, Salminen-Paatero S (2019) Radioecological sample collection in Finland. In: XVIII NSFS Conference “Next Level in Radiation Protection”, Proceedings, Hanaholmen, Espoo/Helsinki, Finland 10 - 14 June 2019. Nordic Society for Radiation Protection. p 109

[CR51] Pontér S, Rodushkin I, Engström E, Rodushkina K, Paulukat C, Peinerud E, Widerlund A (2021). Early diagenesis of anthropogenic uranium in lakes receiving deep groundwater from the Kiruna mine, northern Sweden. Sci Tot Env.

[CR52] Quinto F, Hrnecek E, Krachler M, Shotyk W, Steier P, Winkler SR (2013). Measurements of 236U in ancient and modern peat samples and implications for postdepositional migration of fallout radionuclides. Environ Sci Technol.

[CR53] Rissanen K, Matishov D, Matishov GG (1995) Radioactivity levels in Barents, Petshora, Kara Sea, Laptev and White Sea. In: Strand P, Cooke A (eds.). Environmental Radioactivity in the Arctic. Proceedings of the Second International Conference on Environmental Radioactivity in the Arctic, Oslo, Norway, August 1995. Statens Strålevern, Østerås, Norway, 208–214

[CR54] Rissanen K, Ikäheimonen TK, Matishov D, Matishov GG (1997) Radioactivity levels in fish, benthic fauna, seals and sea birds collected in the Northwest Arctic of Russia. Radioprotection – Colloques vol. 32 No. C2 (International Symposium on Radionuclides in the Oceans - Octeville-Cherbourg, France, 7–11 October 1996), 323–331

[CR55] Rodushkin I, Lindahl P, Holm E, Roos P (1999). Determination of plutonium concentrations and isotope ratios in environmental samples with a double-focusing sector field ICP-MS. Nucl Instrum Methods Phys Res Sect A.

[CR56] Sakaguchi A, Kawai K, Steier P, Quinto F, Mino K, Tomita J, Hoshi M, Whitehead N, Yamamoto M (2009). First results on 236U levels in global fallout. Sci Tot Env.

[CR57] Salbu B, Nikitin AI, Strand P, Christensen GC, Chumichev VB, Lind B, Fjelldal H, Bergan TDS, Rudjord AL, Sickel M, Valetova NK, Føyn L (1997). Radioactive contamination from dumped nuclear waste in the Kara Sea—results from the joint Russian-Norwegian expeditions in 1992–1994. Sci Tot Env.

[CR58] Salminen-Paatero S, Thölix L, Kivi R, Paatero J (2019). Nuclear contamination sources in surface air of Finnish Lapland in 1965–2011 studied by means of ^137^Cs, ^90^Sr, and total beta activity. Environ Sci Pollut R.

[CR59] Salminen-Paatero S, Vira J, Paatero J (2020). Measurements and modeling of airborne plutonium in Subarctic Finland between 1965 and 2011. Atmos Chem Phys.

[CR60] Semenov A (1993) Impact of Chernobyl accident on the Kola Peninsula Radiation Conditions, In: Strand P, Holm E (eds.) Environmental radioactivity in the Arctic and Antarctic. Proceedings of International Conference on Environmental Radioactivity in the Arctic, Kirkenes, Norway, 23–27 August, 1993. Statens Strålevern, Österås, 353–356

[CR61] Steier P, Bichler M, Fifield LK, Golser R, Kutschera W, Priller A, Quinto F, Richter S, Srncik M, Terrasi P, Wacker L, Wallner A, Wallner G, Wilcken KM, Wild EM (2008). Natural and anthropogenic ^236^U in environmental samples. Nucl Instrum Methods Phys Res Sect B.

[CR62] Strand P, Nikitin A, Rudjord AL, Salbu B, Christensen G, Føyn L, Kryshev II, Chumichev VB, Dahlgaard H, Holm E (1994). Survey of artificial radionuclides in the Barents Sea and the Kara Sea. J Environ Radioact.

[CR63] Suksi J, Rasilainen K, Pitkänen P (2006). Variations in 234U/238U activity ratios in groundwater—a key to flow system characterisation?. Phys Chem Earth.

[CR64] Szczypa J, Reszka M (1998) Monitoring of radioactive contamination of the Barents Sea region. Geomorfologia, Wyprawy Geograficzne na Spitsbergen. IV Zjazd Geomorfologów Polskich, UMCS, Lublin, 3–6 czerwca 1998. pp 237–249

[CR65] Tuominen Y, Jaakkola T, Ahmadjian V, Hale ME (1973). Absorption and accumulation of the mineral elements and radioactive nuclides. The Lichens.

[CR66] UNSCEAR (1982) Ionizing radiation: sources and biological effects. United Nations Scientific Committee on the Effects of Atomic Radiation 1982 Report to the General Assembly, With Annexes. https://www.unscear.org/docs/publications/1982/UNSCEAR_1982_Report.pdf. Accessed 3 Jan 2023

[CR67] UNSCEAR (2000) ANNEX C: Exposures to the public from man-made sources of radiation. Sources and Effects of Ionizing Radiation, United Nations Scientific Committee on the Effects of Atomic Radiation UNSCEAR 2000 Report to the General Assembly, with Scientific Annexes. Sources I, pp 157–292. https://www.unscear.org/docs/publications/2000/UNSCEAR_2000_Annex-C-CORR.pdf . Accessed 3 Jan 2023

[CR68] Wallner G, Uguz H, Kern M, Jirsa F, Hain K (2022). Retrospective determination of fallout radionuclides and ^236^U/^238^U, ^233^U/^236^U and ^240^Pu/^239^Pu atom ratios on air filters from Vienna and Salzburg, Austria. J Environ Radioact.

[CR69] Wendel CC, Fifield LK, Oughton DH, Lind OC, Skipperud L, Bartnicki J, Tims SG, Høibråten S, Salbu B (2013). Long-range tropospheric transport of uranium and plutonium weapons fallout from Semipalatinsk nuclear test site to Norway. Environ Int.

[CR70] Yablokov AV (2001). Radioactive waste disposal in seas adjacent to the territory of the Russian Federation. Mar Pollut Bull.

